# Screening of CXC chemokines in the microenvironment of ovarian cancer and the biological function of CXCL10

**DOI:** 10.1186/s12957-021-02440-x

**Published:** 2021-11-18

**Authors:** Weiyuan Li, Ji-Ao Ma, Xun Sheng, Chunjie Xiao

**Affiliations:** School of Medicine, Yunnan University, No.2, Cuihu North Road, Kunming, 650091 Yunnan Province People’s Republic of China

**Keywords:** Ovarian cancer, Chemokines, Angiogenesis, Immune infiltration, Bioinformatics

## Abstract

**Background:**

This study aims to screen and identify the biological functions and prognostic value of CXC chemokines in ovarian cancer (OC) through bioinformatics and molecular biology methods, and to provide data support for the selection of biomarkers and prognostic analysis of OC.

**Methods:**

In this study, GEO, ONCOMINE, GEPIA, cBioPortal, GeneMANIA, Metascape, STRING, TRRUST, and TIMER databases were used to study CXC chemokines. Angiogenesis and T cell killing assay were used to detect the effect of CXCL10 on tumor cell immunity and angiogenesis. Real-time quantitative PCR (qRT-PCR), immunoblotting, and ectopic tumor formation experiments were used to verify the effect of CXCL10 on ovarian cancer tumors.

**Results:**

We found that CXCL1, CXCL10, CXCL11, CXCL13, and CXCL14 were significantly upregulated in OC samples compared with normal tissues. Our data showed that there was a relationship between the expression of CXC chemokines and the infiltration of six types of immune cells significant correlation. In vitro assay confirmed that overexpression of CXCL10 could enhance the killing effect of T cells and inhibit angiogenesis. Further in vivo assay had shown that CXCL10 could affect the progression of ovarian cancer by increasing the expression of cytotoxic T cells and inhibiting angiogenesis.

**Conclusion:**

In conclusion, we hope that our data will provide new insights into the development of immunotherapy and the selection of prognostic markers for patients with OC.

**Supplementary Information:**

The online version contains supplementary material available at 10.1186/s12957-021-02440-x.

## Background

Ovarian cancer (OC) is the sixth most common female cancer in the world, the second largest gynecological malignant tumor, and the deadliest tumor of the human female reproductive system [1]. Although treatments for ovarian cancer had improved, most patients are still at risk of recurrence. In recent years, there was evidence that OC was an immunogenic tumor, and it had been observed that tumor-infiltrating lymphocyte subsets were closely related to the overall survival of patients [2]. With the increasing research of immunotherapy in the treatment of tumors, its efficacy has attracted people’s attention. However, the current specific mechanism is still unclear. Therefore, this study attempts to screen and verify more therapeutic targets and prognostic biomarkers through a combination of bioinformatics and molecular biology.

Chemokines were secreted by tumor cells and other types of cells and regulate anti-tumor immune responses by regulating immune cell transport and other pathways [3]. Current evidence suggests that chemokines could directly and indirectly modulates immunity and modulates tumor immunology and biological phenotypes, thereby affecting angiogenesis, tumor proliferation, metastasis, and patient prognosis [4]. CXC chemokine was an important role of the chemokine family, so it may be a potential therapeutic target and prognostic biomarker for a variety of cancers including OC.

Studies had determined the expression and function of some CXC chemokines in OC, but the current research is still very scarce [5, 6]. In addition, the identification of effective molecules as therapeutic targets and biomarkers of OC still urgently needs our in-depth discussion. Therefore, we conducted comprehensive bioinformatics analysis on the expression and function of CXC chemokines in OC through a variety of databases. Through screening, we found that the differential expression of CXCL10 was the most significant. In vitro and in vivo experiments were used to further verify the role of CXCL10 in OC immunity and angiogenesis. Through the discussion of this study, we try to provide more powerful data support for clinicians to choose appropriate drugs and more accurate prognostic information in the treatment of OC patients.

## Materials and methods

### Gene Expression Omnibus analysis

The data set used is from Gene Expression Omnibus (GEO) database, and the download data format is MINIML (GSE6008 and GSE66957). Box plots are drawn by boxplot; PCA graphs are drawn by R software package ggord. The box plot is implemented by the R software package ggplot2; the heat map is displayed by the R software package pheatmap.

### ONCOMINE

In this study, ONCOMINE (www.oncomine.org) extracted data to evaluate the level of CXC chemokines in OC [7]. The significance threshold is *P* < 0.05, fold change > 2, and the gene ranking is the top 10%.

### Gene expression profiling interactive analysis

In this study, Gene expression profiling interactive analysis (GEPIA)’s “single gene analysis” module was used to analyze the differences in mRNA expression and pathological stages between tumor and normal tissues, as well as the prognostic analysis of CXC chemokines. Use the “Ovarian serous cystadenocarcinoma (OV)” data set. The Kaplan-Meier curve was used for prognostic analysis.

### cBioPortal for cancer genomics

In this study, the cBioPortal (www.cbioportal.org) database was used to analyze the gene changes and co-expression modules of CXC chemokines obtained from TCGA data sources [8]. The OV specimens (TCGA) of 606 cases were analyzed.

### GeneMANIA

We use GeneMANIA (http://www.genemania.org) to analyze CXC chemokine protein and genetic interaction information, co-expression and similarity of protein domains to the submitted genes [9].

### STRING

STRING: functional protein association networks (https://string-db.org/). We performed PPI network analysis on CXC chemokines with different expressions to explore their interactions.

### Metascape

In this study, Metascape databases was used to perform gene ontology (GO) and Kyoto Encyclopedia of Genes and Genome (KEGG) pathway enrichment analysis on 50 genes related to CXC chemokines [10].

### TRRUST

The trust (https://www.grnpedia.org/trrust/) database contains 8444 transcription factor (TF) target regulatory relationships of 800 human transcription factors [11]. We use this data to predict the transcription factors of CXC chemokines.

### Immune infiltration analysis

We use the TIMER (https://cistrome.shinyapps.io/timer/) platform for immune infiltration analysis [12]. In this study, the “gene module” and “survival module” were used to evaluate the correlation between CXC chemokine levels and immune cell infiltration and the correlation between clinical results and immune cell infiltration and CXC chemokine expression.

### Cell culture

SK-OV-3 and VE cells were obtained from Procell company (Wuhan, China). All cell lines were cultured in DMEM medium containing 10% FBS and 1% double antibodies. All the cells were incubated at the condition of 37 °C, 5% CO_2_.

### Plasmid construction and cell transfection

On the GenePharma (Shanghai, China), the transfected materials si-CXCL10 and CXCL10 were purchased. Twenty-four hours before transfection, SK-OV-3 cell in the exponential phase were digested by pancreatin and made into cell suspension. Si-CXCL10 and CXCL10 were used for the in vitro assay. After trypsinization from flasks, cells were cultured in six-pore plates, incubated at 37 °C with 5% CO_2_ for 18–24 h. Three hours before transfection, cells at about 80–90% confluency were changed to the serum and antibiotic-free media. Then, cells were transfected using Lipofectamin 2000 reagent (Life Technologies, Gaithersburg, MD, USA) referring to manufacturer’s instructions and incubated at the same conditions as above for 48 h. In addition to the in vivo experiment, based on the manual, concentrated lentiviral solutions of CXCL10 were mixed with two nutrient solution contains ID8 cell, respectively. Finally, the cells were digested by pancreatin and injected into mice after incubation for 48 h.

### T cell-mediated cells killing assay

Refer to reports [13], through the CCK8 test and apoptosis assay to test the T cell killing assay. Then SK-OV-3 cells and T cells were co-cultured for 24 h, and then used for related treatment. The cell viability was analyzed by a microplate reader at 450 nm.

### Cell proliferation ability test

For CCK-8 assay, we employed Cell Counting Kit (Yeasen, China) to determine the viability of cells. The optical absorbance was measured at 450 nm.

### Flow cytometry

The treated cells (1 × 106) were seeded in 6-well plates a. After 48 h of treatment, cells were harvested and centrifuged to remove the supernatant. To perform apoptosis assays, cells were resuspended in binding buffer and stained with Annexin V and PI (BestBio, China). Finally, a flow cytometer (Beckman Coulter, USA) was used to assess cell apoptosis.

### Angiogenesis assay

In this study, 50 μL/well of Matrigel Matrigel was added to a 96-well plate, avoiding air bubbles when added, and placed in a cell incubator for 60 min to solidify, and 100 μL of cell suspension was added to each well (the tumor cell culture medium was treated with different treatments). Then we put it into a cell incubator for routine culture after making the mark, and take a photo with a microscope after incubating for 24 h. The tube formation was visualized under an inverted microscope. Enclosed networks of tube structures from three randomly chosen fields were recorded under a Nikon microscope.

### Animal and tumor models

16 BALB/c mice aged 3–5 weeks were purchased from Charles river Laboratories (Beijing, China). All animals are kept in a constant temperature, constant humidity and no specific pathogen level animal center. Guarantee their unlimited water and diet. All animal studies were approved by the Animal Ethical and Welfare Committee of Yunnan University. The animals were randomly divided into 2 groups (NC group, CXCL10 group, *n* = 6) and subcutaneously inoculated in the right flank with 5 × 10^5^ ID8 cells. Tumor volume was calculated using the formula: π/6 × length×width^2^. The tumor size was measured every 3 days. On day 30, the mice were killed, and the tumors were dissected and weighed.

### Real-time PCR

Total RNA was extracted by the TRIzol (Ambion, USA). All qRT-PCR processes were accomplished using the SYBR Green qPCR Master Mix (MedChem Express, NJ, USA). The results were calculated by the 2^-ΔΔCt^ method. GAPDH was used for normalization as a control. Primers were shown in Supplementary Table 1.

### Hematoxylin-eosin, Ki67, and TUNEL staining

In this study, the tumor tissues of mice in each treatment group were paraffin-embedded and sliced, and then the tissues were stained with the Ki-67 immunohistochemistry kit and Tunel analysis kit referring to the manufacturer’s method. Hematoxylin and eosin were used to stain paraffin sections of mouse tumor tissues after deparaffinization and dehydration. Finally, observe the cell morphology under a microscope. We used ImageJ software to quantify the number of KI67, TUNEL and CD8-positive cells.

### Immunohistochemistry and immunofluorescence

The immunohistochemistry (IHC)-stained tissue sections were reviewed under microscope by 3 pathologists who were blinded to the clinical parameters, and scored independently according to the intensity of cellular staining and the proportion of stained tumor cells. The VEGFA and COX2 proteins were immunohistochemically stained yellowish to brown in the cytoplasm, and displayed all or none mode in tumor tissues. The degree of immunostaining of indicated proteins was evaluated and scored by 3 independent observers. Briefly, each sample was scored according to staining intensity (no staining = 0; weak staining = 1; moderate staining = 2; strong staining = 3) and the number of stained cells (0% = 0; 1–25% = 1; 26–50% = 2; ≥ 51% = 3). The staining index (SI) was calculated as the product of staining intensity × percentage of positive tumor cells, result in scores of 0, 1, 2, 3, 4, 6, and 9. The microvessel density (MVD) was evaluated by CD34 immunofluorescence staining. MVD was calculated as the average count of microvessel in the 4 hot spots at high magnification (200×).

### Western blot

First, we extract proteins from cells and tissues in different treatment groups, and then quantify each group of proteins with the BCA kit. Secondly, after electrophoresis of different groups of proteins and transfer to the membrane, the primary antibodies of human and mouse VEGFA, CD34, COX2, and GAPDH (Abcam) were incubated in the membrane overnight at 4 °C and then combined with the secondary antibody. Finally, use an enhanced chemiluminescence kit to check the binding of the antibody.

### Statistical analysis

All data in this study used Excel for data database construction and input, and SPSS 22.0 and GraphPad prism 9 software for data statistics. The measurement data were expressed in the form of mean ± SEM, the comparison between multiple groups is compared by one-way analysis of variance (ANOVA), and the Tukey’s was used for pairwise comparison. Inspection level α = 0.05.

## Result

### The level of CXC chemokines expression in OC patients

**Fig. 1:**
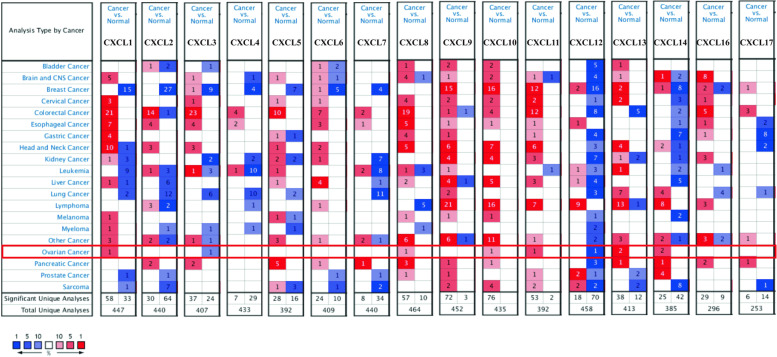
The expression of CXC chemokine mRNA in OC patients. The picture shows the expression of CXC chemokine mRNA that is statistically significant. The number of data sets that express upregulation (red) and express down-regulation (blue)

Transcriptional levels of 16 CXC chemokines (excluding CXCL15) in OC and ovarian tissues were analyzed using Oncomine database. It was found that the transcriptional levels of CXCL1, CXCL8, CXCL10, CXCL11, CXCL13, and CXCL14 in OC tissues were significantly increased. The transcription levels of CXCL3 and CXCL12 were lower than those in normal tissues (Fig. 1 and Supplementary Fig. 1). In addition, we have also demonstrated this phenomenon in a large body of literature (Table 1) [14–17, 20, 21]. As shown in Table 1, among the up-regulated CXC chemokines, CXCL10 had the highest differential expression multiple, and similarly, CXCL12 had the highest differential expression multiple when downregulated.

**Table 1:** The mRNA levels of CXC chemokines in different types of OC tissues and normal tissues at transcriptome level (ONCOMINE)

TLR	Type	Fold change	*t* test	*P* value	References
CXCL1	Ovarian Serous Adenocarcinoma	2.753	4.093	0.004	[14]
CXCL3	Ovarian Serous Adenocarcinoma	− 5.279	− 4.912	0.000	[15]
CXCL8	Ovarian Serous Adenocarcinoma	2.455	2.562	0.015	[16]
CXCL10	Ovarian Serous Adenocarcinoma	9.290	5.697	0.000	[17]
CXCL11	Ovarian Serous Adenocarcinoma	2.995	9.548	0.000	TCGA
CXCL12	Ovarian Serous Adenocarcinoma	− 29.039	− 13.783	0.000	[17]
CXCL13	Ovarian Serous Adenocarcinoma	2.245	12.237	0.000	[18]
CXCL14	Ovarian Serous Adenocarcinoma	2.762	6.752	0.000	[19]

**Fig. 2:**
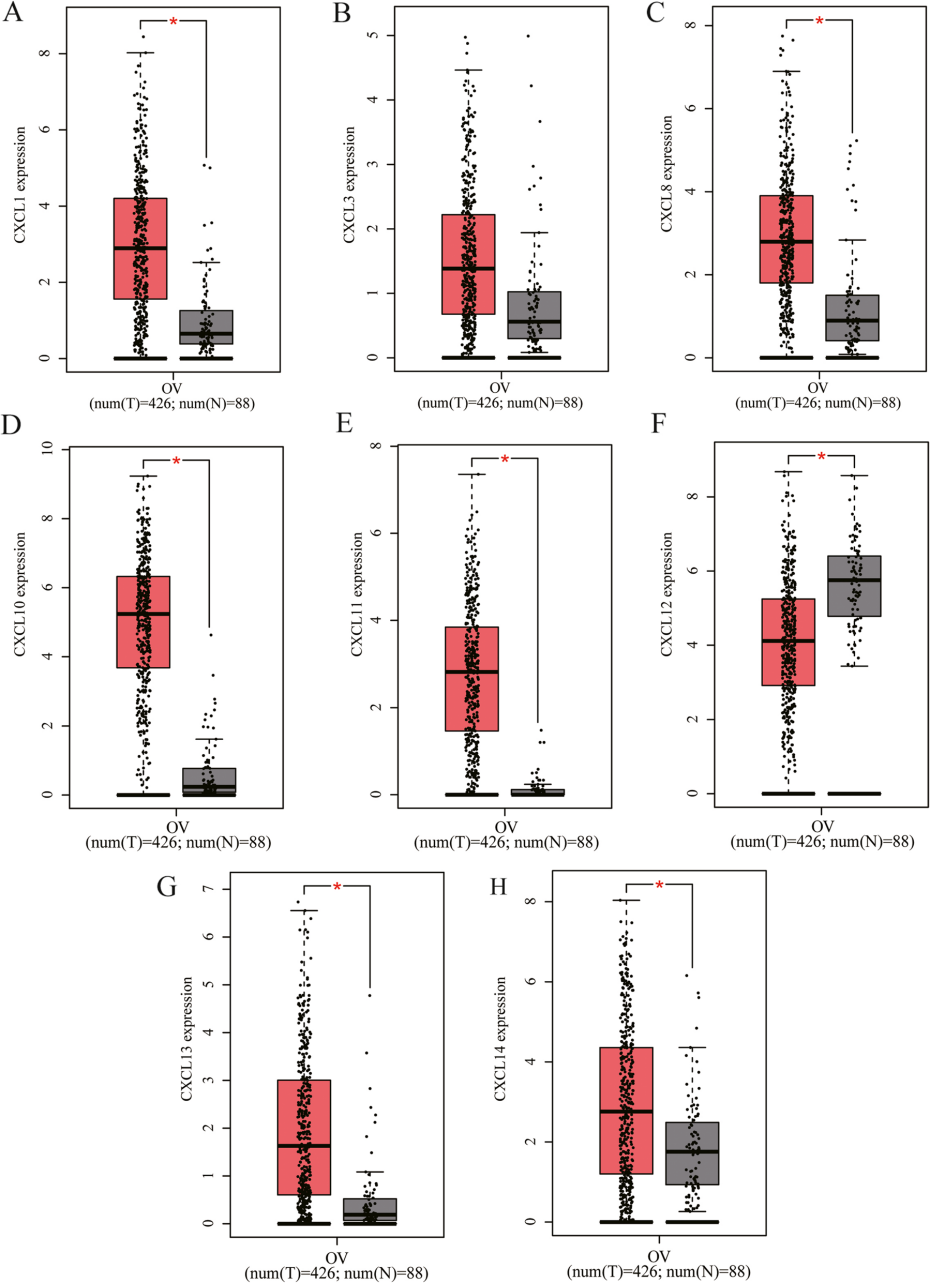
GEPIA database verifies the expression of CXC chemokines. In this study, the GEPIA database used TCGA data to verify the expression of CXC chemokines. *p < 0.05 vs. normal

In addition, we used TCGA data to further verify the level of CXC chemokines expression through the GEPIA database. As expected, we also saw CXCL1, CXCL8, CXCL10, CXCL11, CXCL13, and CXCL14 in tumor tissues were upregulated in the results of TCGA data and the difference was statistically significant (*p* < 0.05). In the TCGA database, only the downregulation of CXCL12 was different, while the expression of CXCL3 was not statistically different (Fig. 2).

**Fig. 3:**
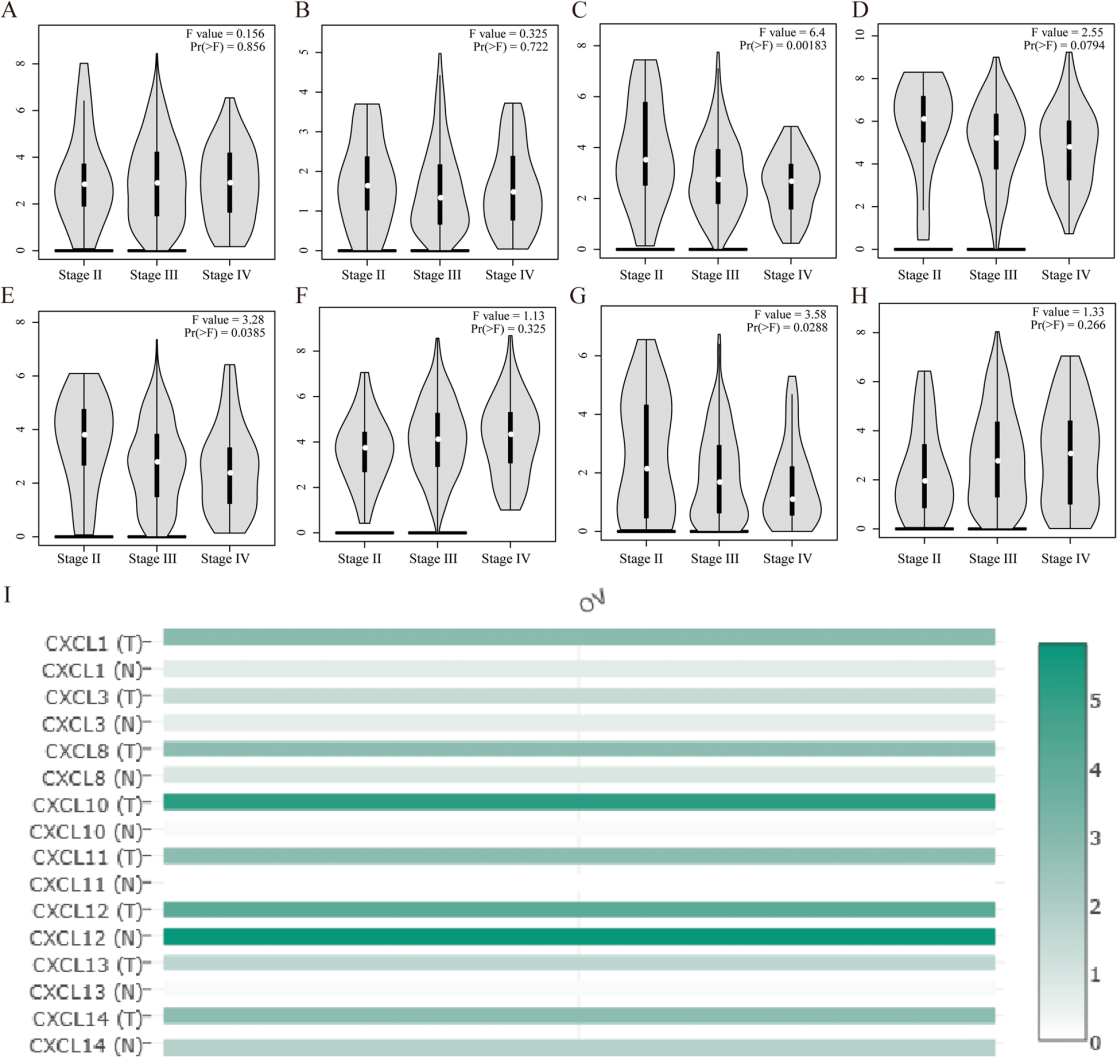
GEPIA database verifies the expression of CXC chemokines in different pathological stages and tissues. Use TCGA data to analyze the expression differences of different chemokines (**A** CXCL1, **B** CXCL3, **C** CXCL8, **D** CXCL10, **E** CXCL11, **F** CXCL12, **G** CXCL13, **H** CXCL14) in different pathological stages of OV. **I** In OC patients, the expression level of different CXC chemokines is that the color with high expression is darker, and the color with low expression is lighter

Then, we examined the correlation between CXC chemokines and the pathological stage of OC patients and found that the expression of CXCL8 (*P* = 0.002), CXCL11 (*P* = 0.039), and CXCL13 (*P* = 0.029) gradually increased with the aggravation of the pathological stage Decline, showing a trend of negative correlation (Fig. 3A–H). Based on the above results, we seem to have discovered an interesting phenomenon. The expressions of these three CXC chemokines all gradually decrease with the progress of the pathological stage. Further, we analyzed the expression of these chemokines using the data of the TCGA database in the GEPIA database and found that the expression of CXCL10 was the highest and the difference was significant (Fig. 3I).

### Analysis of the prognostic value of CXC chemokines in OC patients

**Fig. 4:**
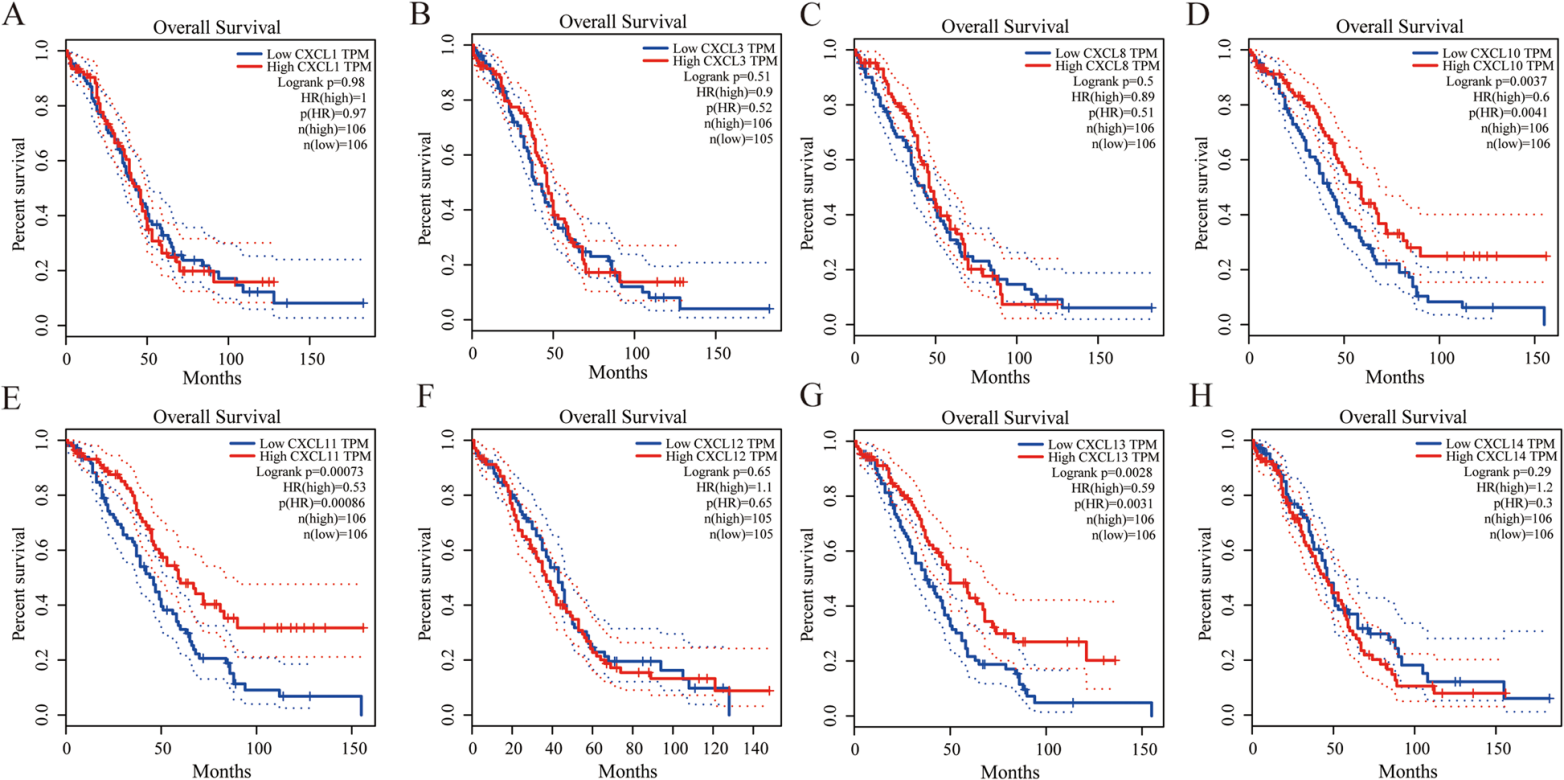
The prognostic value of different expressed CXC chemokines in OC patients in the overall survival curve. The overall survival curve of **A** CXCL1, **B** CXCL3, **C** CXCL8, **D** CXCL10, **E** CXCL11, **F** CXCL12, **G** CXCL13, and **H** CXCL14 in OC. All survival data are derived from TCGA data in the GEPIA database

The overall survival curve is shown in Fig. 4. The high expression of CXCL10 (*P* = 0.004), CXCL11 (*P* = 0.000), and CXCL13 (*P* = 0.003) can improve the survival rate of OC patients. On the other hand, the value of differentially level CXC chemokines in disease-free survival of OC patients was also evaluated. Here, we only found that the high expression of CXCL13 has a promoting effect on disease-free survival (Supplementary Fig. 2).

### CXC chemokine expression, co-expression, gene network, and interaction analysis in OC patients

**Fig. 5:**
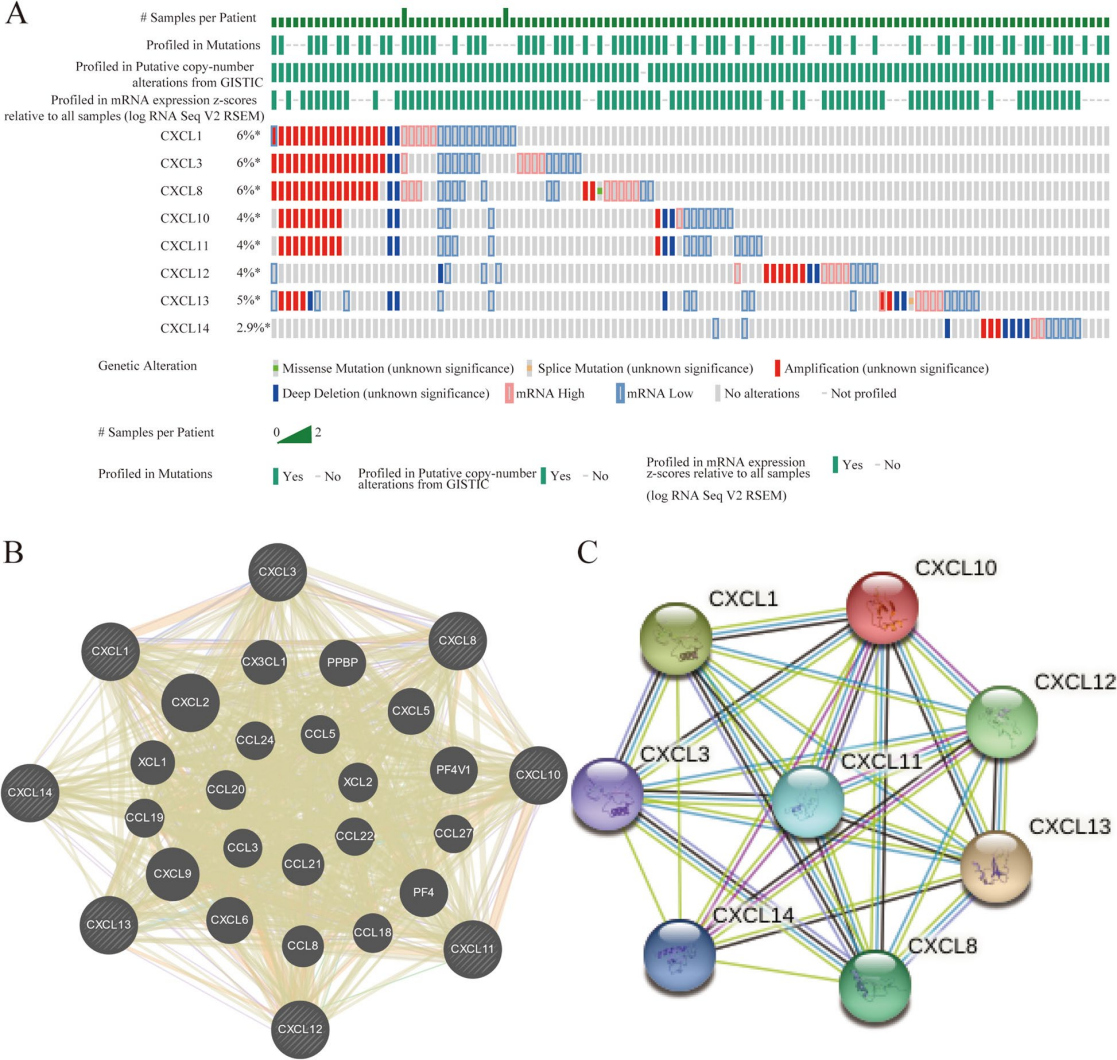
CXC chemokine expression, gene network and interaction analysis in OC patients. **A** The changes of CXC chemokines expressed in OC. **B**, **C** The protein-protein interaction network of different CXC chemokines

The temporary data set of TCGA was used to analyze the genetic changes of CXC chemokines. As a result, in the OC samples that were queried, the changes of CXCL1, CXCL3, CXCL8, CXCL10, CXCL11, CXCL12, and CXCL13 were 6, 6, 6, 4, 4, 4, 5, and 2.9%, respectively (Fig. 5A). Here, amplification accounted for most of the reasons for the change, which also found the reason for the increase of some CXC chemokines. The GeneMANIA database shows that the functions of differentially expressed CXC chemokines were mainly related to cell chemotaxis and chemokine activity related (Fig. 5B). In addition, the results of PPI network analysis of STRING database show that these CXC chemokines interact as shown in the figure (Fig. 5C).

### Analysis of GO and pathway of CXC chemokines in OC patients

First, we obtained 50 genes related to CXC chemokines by referring to previous research results. Then we used Metascape to analyze their functions. Supplementary Fig. 3A shows the top 10 richest GO categories using Metascape. Among the biological process (BP) category, regulation of cytosolic calcium ion concentration pathway, G protein coupled receptor signaling pathway, regulation of secretion, calcium ion transport, and response to wounding were related to the occurrence and development of OC. In the molecular function (MF) category, we found that these genes play a role in both chemokine receptor binding and cytokine receptor binding activity (Supplementary Fig. 3B). Side of membrane, dendritic tree, heterotrimeric G-protein complex, cell body, endocytic vesicle, platelet alpha granule lumen, focal adhesion, vacuum, midbody, and postsynaptic membrane are the 10 most enriched OC categories in cells (Supplementary Fig. 3C). In addition, we also performed KEGG pathway analysis. We found that the chemokine signaling pathways, cytokine-cytokine receptor interactions, and cancer pathways were closely related to tumorigenesis and the progression of OC (Supplementary Fig. 3D).

### Transcription factor targets of CXC chemokines in patients with OC

We use the TRRUST database to explore the transcription factor target information of the differentially expressed CXC chemokines. As shown in Table 2, we found that the transcription factors RELA, SP1, and NFKB1 are involved in the regulation of the biological effects of CXC chemokines. Among them, RELA and NFKB1 have been discovered as key transcription factors of CXCL1, CXCL8, CXCL10, and CXCL12, while SP1 was the key transcription factor of CXCL1 and CXCL14.

**Table 2:** Key regulated factor of CXC chemokines in OC (TRRUST)

Key TF	Description	Gene	FDR	*P* value
RELA	v-rel reticuloendotheliosis viral oncogene homolog A (avian)	CXCL1, CXCL8, CXCL10, CXCL12	6.50E-06	4.22E-06
NFKB1	Nuclear factor of kappa light polypeptide gene enhancer in B cells 1	CXCL1, CXCL8, CXCL10, CXCL12	6.50E-06	4.33E-06
SP1	Sp1 transcription factor	CXCL1, CXCL14	0.0158	0.0158

### Correlation between CXC chemokines and immune cell infiltration in OC patients

**Fig. 6:**
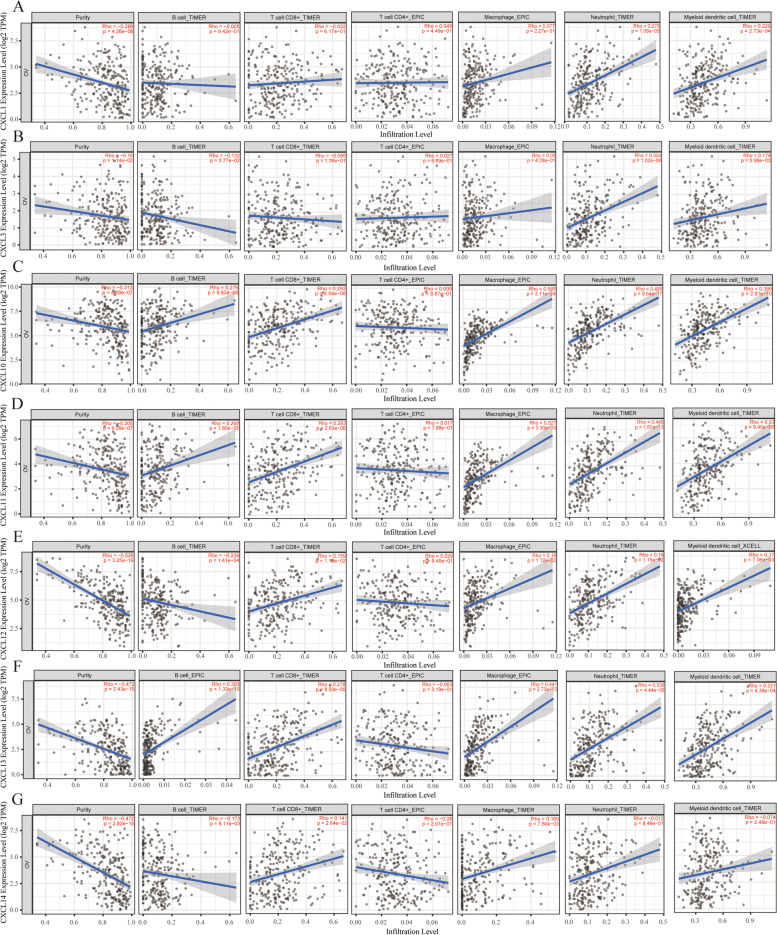
Correlation between CXC chemokines and immune cell infiltration in OC patients. The correlation between the abundance of immune cell and the expression of **A** CXCL1, **B** CXCL3, **C** CXCL8, **D** CXCL10, **E** CXCL11, **F** CXCL12, **G** CXCL13, and **H** CXCL14 in OC

This study used the TIMER database to analyze the correlation between CXC chemokines and immune cell infiltration. CXCL1 was positively correlated with neutrophils (Cor = 0.275, *P* < 0.05) and dendritic cells (Cor = 0.229, *P* < 0.05). Similarly, CXCL3 was positively correlated with neutrophils (Cor = 0.304, *P* < 0.05) and dendritic cells (Cor = 0.174, *P* < 0.05), in addition to B cells (Cor = − 0.132, *P* < 0.05). There was a negative correlation. CXCL10, CXCL11, and CXCL13 were all positively correlated with B cells, CD8^+^ T cells, macrophages, neutrophils, and dendritic cells. However, the immune infiltration trend of CXCL12 and CXCL14 was similar. They are all positively correlated with macrophages, neutrophils and dendritic cells, but negatively correlated with B cells (Fig. 6) (*P* < 0.05).

### The GEO database detects the expression of CXC chemokines in OC

**Fig. 7:**
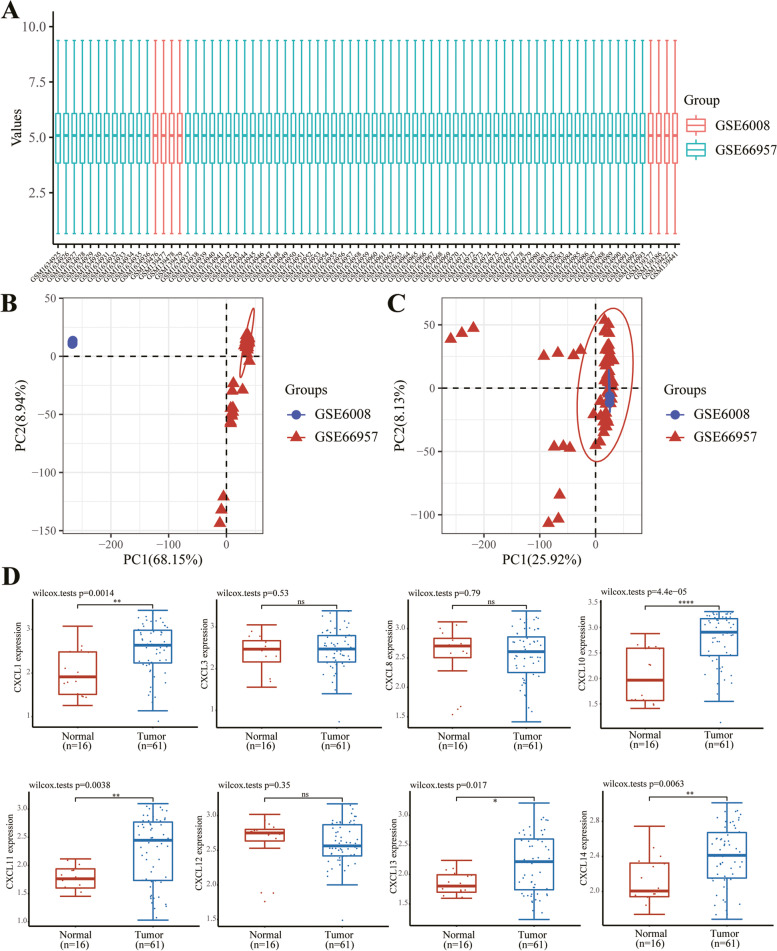
The GEO database detects the expression of CXC chemokines in OC. **A**. Standardization of each sample data. **B**, **C** PCA analysis shows the data changes before (**B**) and after (**C**) processing, and the intersection of the processed data sets can be used for further analysis. **D** The expression distribution of CXC gene in tissues, where the horizontal axis represents different groups of samples, the vertical axis represents the gene expression distribution, where different colors represent different groups, and the upper left corner represents the significance p-value test method

Through the above analysis, we have discovered the role of different CXC chemokines in OC. To further verify the expression of different CXC chemokines, we selected the data of two GEO samples of GSE6008 and GSE66957 to verify the expression of them. First, we standardized the data (Fig. 7A–C). After data processing, we also found that the expression of CXCL1, CXCL10, CXCL11, CXCL13, and CXCL14 was significantly increased in tumor tissues, and the difference between CXCL10 was the most significant (Fig. 7D).

### The effect of CXCL10 on tumor and angiogenesis

**Fig. 8:**
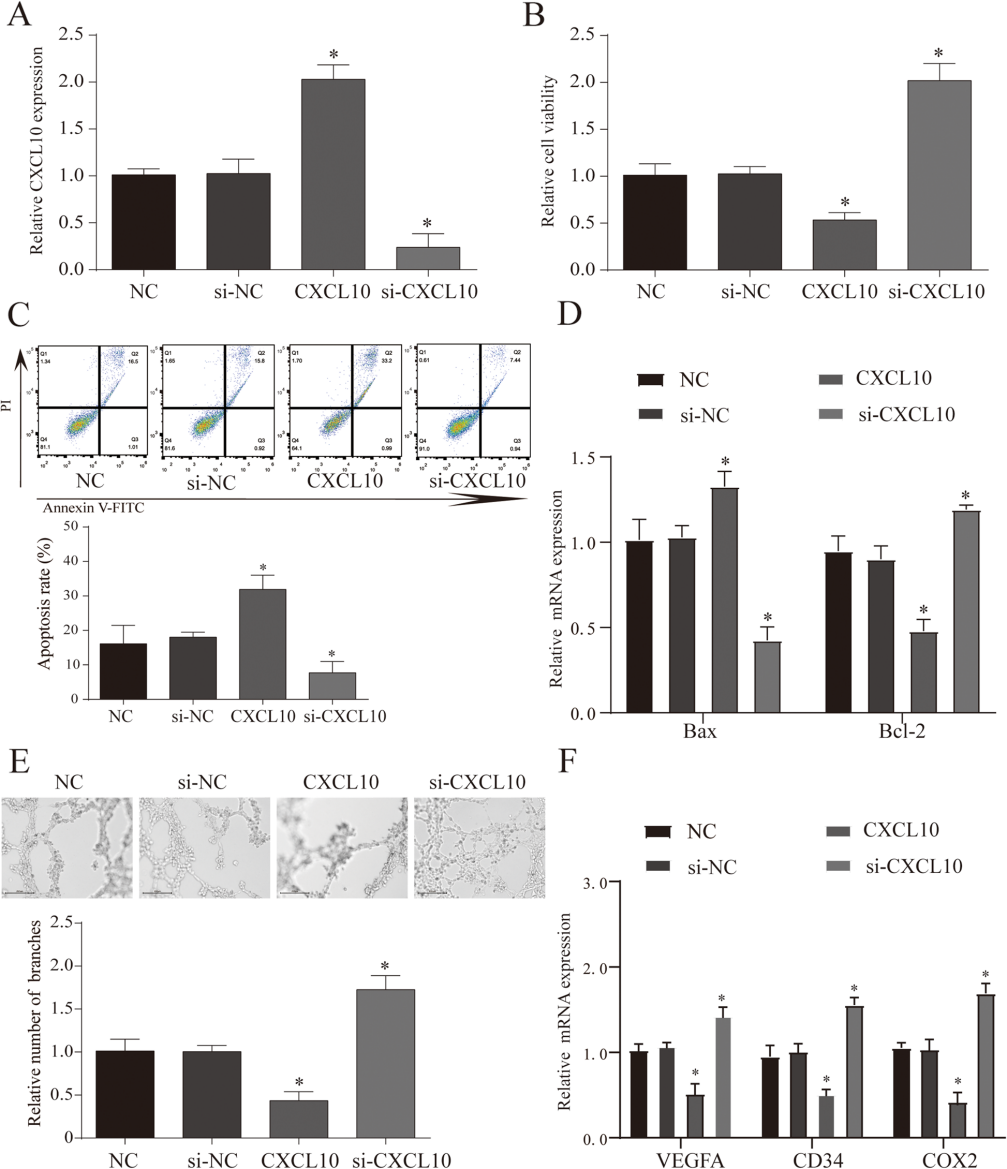
The effect of CXCL10 on cellular immunity and angiogenesis. **A** The expression of CXCL10 mRNA in each treatment group. **B**, **C** The cell viability and apoptosis in each treatment group. **D** Cell viability and apoptosis in each treatment group. **E** An in vitro Matrigel tube formation assay was performed to evaluate the angiogenic effect of CXCL10, and representative micrographs images are shown at × 200. The number of branches were analyzed, and the data represent the means ± SEM of triplicates. **F** MRNA and protein expression of angiogenesis markers (VEGFA, COX2, and CD34) in different treatments. *NC* normal control group, *si*-*NC* inhibitor control group, *si*-*CXCL10* suppress CXCL10 expression group, *CXCL10* overexpression CXCL10 group, *p < 0.05 vs. NC group

**Fig. 9:**
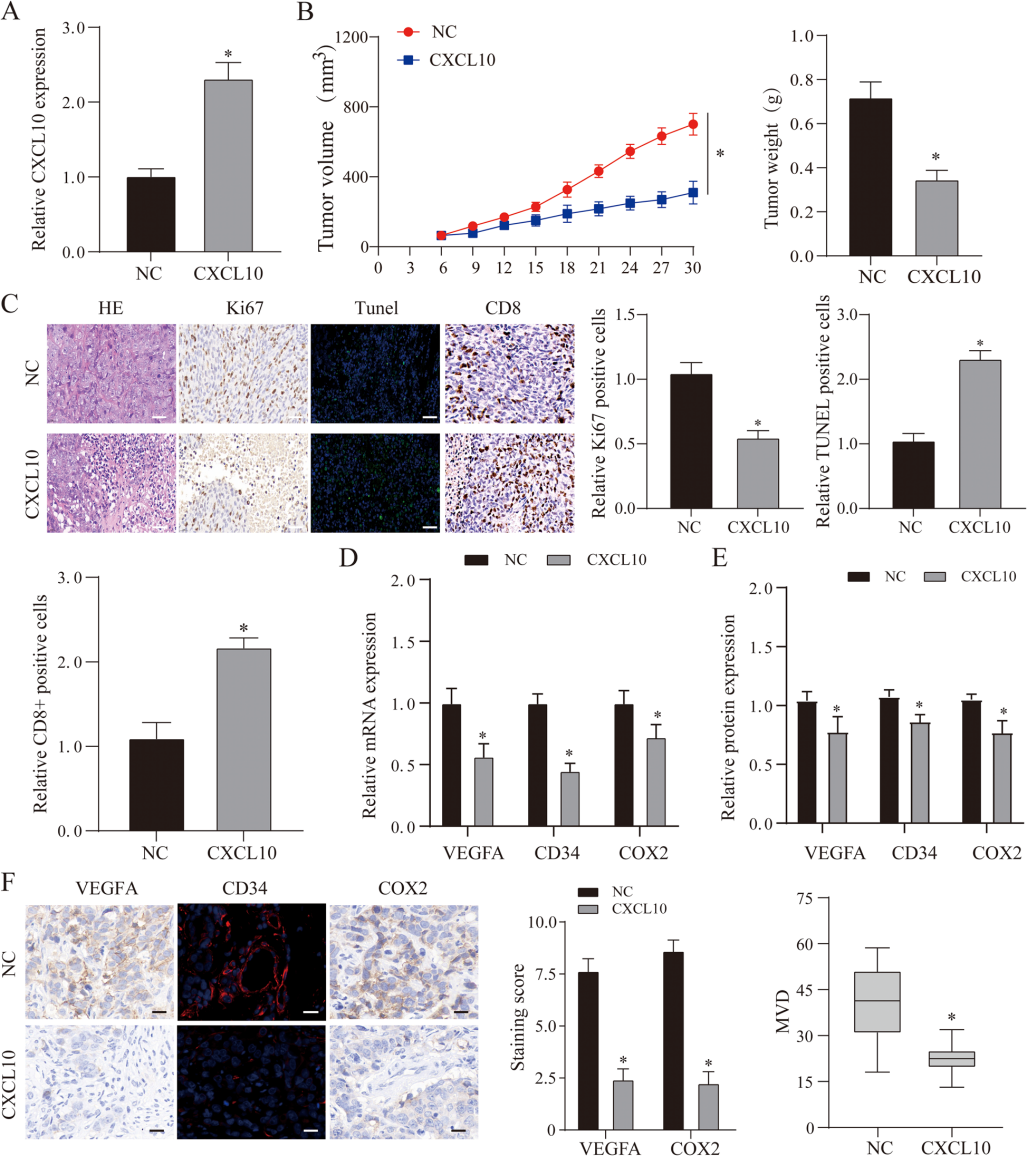
The effect of CXCL10 on tumor and angiogenesis. **A** QRT-PCR verified the expression of CXCL10 mRNA. **B** Tumor size and volume in each treatment group. **C** Tumor tissue HE, KI67, TUNEL, and CD8^+^ cell staining results. We use Image J to quantitatively analyze the number of KI67, TUNEL, and CD8-positive cells, and all data are expressed in the form of mean + SEM. **D**, **E** MRNA and protein expression of angiogenesis markers (VEGFA, COX2, and CD34) in different treatments. **F** IHC scores of COX2 and VEGFA in tissues was compared with each group. MVD was calculated as the average measurement of 10 random high-power fields (20×). *NC* normal control group, *si*-*NC* inhibitor control group, *si*-*CXCL10* suppress CXCL10 expression group, *CXCL10* overexpression CXCL10 group, *p < 0.05 vs. NC group

Through the above bioinformatics analysis, we found that CXCL10 may play an important role in the occurrence and development of OC. In addition, referring to other studies, we found that CXCL10 has a different trend in other tumor studies. In this study, we found that it is correlated with immune infiltration and patient survival. First, we use the T cell killing test to determine the effect of CXCL10 on immunity. We co-cultured tumor cells with different CXCL10 interference with T cells and found that overexpression of CXCL10 can effectively enhance tumor suppression and apoptosis caused by T cells, and this phenomenon was reversed when CXCL10 was inhibited (Fig. 8A–D). Furthermore, we selected an animal model of ovarian cancer that overexpressed CXCL10 for verification. The results showed that the tumor volume and weight after overexpression of CXCL10 were smaller than that of the NC group (Fig. 9A, B) (*P* < 0.05). After further staining the tumor tissue, it was found that the tumor proliferation ability after overexpression of CXCL10 was weakened, and the cell apoptosis was significantly increased (Fig. 9C). Furthermore, after staining the tumor tissue with CD8, we found that the number of CD8 positive cells in the tumor overexpressing CXCL10 was more than that in the NC group. This result shows that CXCL10 can promote the killing effect of cytotoxic T cells on tumor cells, which is similar to the result of the above analysis (Fig. 9C). Previously shown in other tumors, CXCL10 can effectively inhibit tumor angiogenesis. First of all, when we used differently treated tumor cell culture media to process VE cells to detect angiogenesis, we found that overexpression of CXCL10 could inhibit angiogenesis, while the results of inhibiting CXCL10 are the opposite (Fig. 8E, F). In addition, we further tested angiogenesis markers and immunochemical staining methods to verify this phenomenon (Fig. 9D–F) (*P* < 0.05). In summary, we found that CXCL10 can inhibit the growth of ovarian cancer by increasing immune killing and inhibiting angiogenesis.

## Discussion

A large number of studies had shown that CXC chemokines play an important role in tumorigenesis, proliferation and apoptosis [18]. However, the biological function and prognostic value of CXC chemokines in OC remain unclear. This study was the first to screen CXC chemokine markers in ovarian cancer through bioinformatics methods, and verified through in vivo and in vitro experiments that CXCL10 influences the process of ovarian cancer immune response and angiogenesis. Our data attempts to provide a basis for future ovarian cancer immunotherapy and biomarker selection.

First, we tested the expression of CXC chemokine in tumors and its correlation with the expression in different pathological stages. The results showed that expression of CXCL1, CXCL10, CXCL11, CXCL13, and CXCL14 were significantly increased in OC tissues. These results are obtained by re-arguing with two GEO database samples. They were similar to the results of previous literatures. In addition, we found that increased CXCL10, CXCL11, and CXCL13 expression increased survival in patients with OC with tumor progression. These data suggest that they may play an important role in the OC process. Although the effect of CXC chemokine on ovarian cancer has been reported before [19], the specific role and mechanism of CXC chemokine are still unclear.

Due to the phenomenon of differential expression of CXC chemokines, we conducted a comprehensive analysis on the molecular characteristics of differential expression of CXC chemokines. The results showed that real amplification was the main reason for this change, which also found the reason for the increase of some CXC chemokines. Tumorigenesis is complex and multifaceted, and genetic changes play an important role in this process. Zeng et al.’s study found a synergistic effect of CXC chemokines in renal cancer [18].

Then, we used the Metascape database to perform GO and KEGG pathway enrichment analysis on the verified differentially expressed CXC chemokines to determine their possible biological functions. Through the above data, we confirmed that these genes may function through chemokine signaling pathways, cytokine-cytokine receptor interactions and other pathways. A lot of reports had shown that the chemokine signaling pathways may play an important role in the proliferation, aging, angiogenesis, and immune escape of cancers. This also provides us with further molecular biology experiments. In summary, the differential expression of CXC chemokines may be a potential drug target for the treatment of OC patients.

Rela phosphorylation has been shown to be involved in disease progression, particularly in inflammatory disease and cancer, by regulating NF-κB signal pathway [22]. However, NFKB1 is an inhibitor of inflammation and cancer and plays a tumor-inhibiting role by reducing abnormal activation of the NF-κB signaling pathway [23]. The role of SP1 in ovarian cancer has been discovered by people. For example, Zhang et al. found that SP1-12LOX axis plays a role in the progression and drug resistance of ovarian cancer [24]. Other studies have shown that it can affect the progression of ovarian cancer by affecting the generation of long non-coding RNA [25]. These data support the interpretation of the effect of CXC chemokines in OC.

There is increasing evidence that immune cell infiltration can influence tumor progression and recurrence and become an important determinant of immunotherapeutic response and clinical outcome [26].CD4^+^ T cells recognize cancer antigens, while macrophages may be involved in tumor inhibition [27]. In this study, we found that the level of CXC chemokines was related to the infiltration of immune cells. The correlation indicates that they reflect the effective tumor immune status and provide data for immunotherapy.

Finally, we chose two GEO data (GSE6008 and GSE66957) to try to further study different CXC chemokines. According to the multiples of differential expression, the expression level of tumor tissues, and the importance of biological functions in tumors, CXCL10 was selected for in-depth discussion. The study found that CXCL10 could inhibit tumor growth by constructing a model of ovarian cancer with overexpression of CXCL10. As previously reported, CXC10 plays a different role in different types of tumors [28]. As we predicted using the database, we first verified the immune effect of CXCL10 through T cell killing experiments and found that overexpression of CXCL10 can promote the killing of tumor cells by T cells. Furthermore, we used immunohistochemistry to detect the overexpression of CXCL10 in tumor models and found that overexpression can cause more cytotoxic T cells to infiltrate the tumor tissue.

This study is the first to demonstrate its ability to inhibit tumor growth in ovarian cancer. Further, some studies have found that CXCL10 can exert tumor inhibition by inhibiting angiogenesis in colorectal cancer, lung cancer, cervical cancer, and other tumors [29, 30]. Therefore, we selected CD34, COX2, and VEGFA to detect the markers of angiogenesis, and found that the overexpression of CXCL10 in OC tissue could effectively inhibit angiogenesis. This phenomenon has been further verified by the angiogenesis experiment of VE cells treated with the culture medium after in vitro tumor cell treatment. This may be one of the reasons for its tumor-inhibiting effect.

This study was the first to explore the expression and biological functions of a variety of CXC chemokines in OC. Through in-depth bioinformatics analysis, we selected CXCL10 as the object of further research. We also verified the role of CXCL10 in the OC process. OC is currently one of the deadliest gynecological malignancies. Surgery and chemotherapy are the main treatments for ovarian cancer. In the past two decades, immunotherapy has developed rapidly [31]. In the future, this approach may change the main development direction of various cancer therapies. However, the current immunotherapy response rate for ovarian cancer patients is still moderate, but with the development of new treatments such as TCR engineered T cells, this situation will be changed [32].

## Conclusions

Our results are the first to find that CXCL10 can not only activate the killing effect of T cells in the body on tumors, but also we have seen its impact on angiogenesis on the other hand. Current studies have found that using immunotherapy as an adjuvant therapy, combined with anti-angiogenic drugs, can significantly improve the efficiency of treatment. This view fits the conclusion of this research. Nevertheless, we still lack clinical sample data and in-depth discussion of its mechanism, which will be one of the main directions of our future research. In short, our data discovered the effects of different CXC chemokines on ovarian cancer and conducted in-depth verification of CXCL10 in two aspects: immune regulation and angiogenesis. This study will provide novel insights for the selection of immunotherapy targets and prognostic markers for OC patients.

## Supplementary Information

**Additional file 1: Supplementary table 1**. Primer sequence.

**Additional file 2: Supplementary Figure 1**. Oncomine database analysis of the expression of different chemokines. **Supplementary Figure 2**. The prognostic value of different expressed CXC chemokines in OC patients in the disease free survival curve. **Supplementary Figure 3** GO and pathway analysis of CXC chemokines related genes using Metascape database.

## Data Availability

All data generated or analyzed during this study are included in this published article.

## Abbreviations

OC: Ovarian cancer
GO: Gene ontology
KEGG: Kyoto Encyclopedia of Genes and Genome
TF: Transcription factor
HE: Hematoxylin-eosin
WB: Western blot
OV: Ovarian serous cystadenocarcinoma
