# Supplementary material for: Screening of CXC chemokines in the microenvironment of ovarian cancer and the biological function of CXCL10
Source: World J Surg Oncol. 2021 Nov 18;19:329. doi: 10.1186/s12957-021-02440-x (PMC8600898; doi:10.1186/s12957-021-02440-x)
Supplement: Supplementary file 1 — Additional file 1: Supplementary table 1. Primer sequence. [file 12957_2021_2440_MOESM1_ESM.docx]

**Supplementary table 1. Primer sequence**

|  | **Primer Sequence** |
| --- | --- |
| CXCL10 forward | 5′-AGGGGAGTGATGGAGAGAGG-3′ |
| CXCL10 reverse | 5′-TGAAAGCGTTTAGCCAAAAAAGG-3′ |
| VEGFA forward | 5΄-ACAGAACGATCGATACAGAA-3΄ |
| VEGFA reverse | 5΄-AAAGATCATGCCAGAGTCTC-3΄ |
| CD34 forward | 5’-GGAGCAGGCTGATGCTGATG-3’ |
| CD34 reverse | 5’-ATCCCCAGCTTTTTCAGGTCAGAT-3’ |
| COX2 forward | 5′-GGGTGTCCCTTCGCCTCTTT-3′ |
| COX2 reverse | 5′-GTTGCCGGTATCTGCCTTCA-3′ |
| GAPDH forward | 5′-CCAGGGCTGCCTTCTCTTGT-3′ |
| GAPDH reverse | 5′-CCAGCCTTCTCCATGGTGGT-3′ |
