# Supplementary material for: Screening of CXC chemokines in the microenvironment of ovarian cancer and the biological function of CXCL10
Source: World J Surg Oncol. 2021 Nov 18;19:329. doi: 10.1186/s12957-021-02440-x (PMC8600898; doi:10.1186/s12957-021-02440-x)
Supplement: Supplementary file 2 — Additional file 2: Supplementary Figure 1. Oncomine database analysis of the expression of different chemokines. Supplementary Figure 2. The prognostic value of different expressed CXC chemokines in OC patients in the disease free survival curve. Supplementary Figure 3 GO and pathway analysis of CXC chemokines related genes using Metascape database. [file 12957_2021_2440_MOESM2_ESM.docx]

**
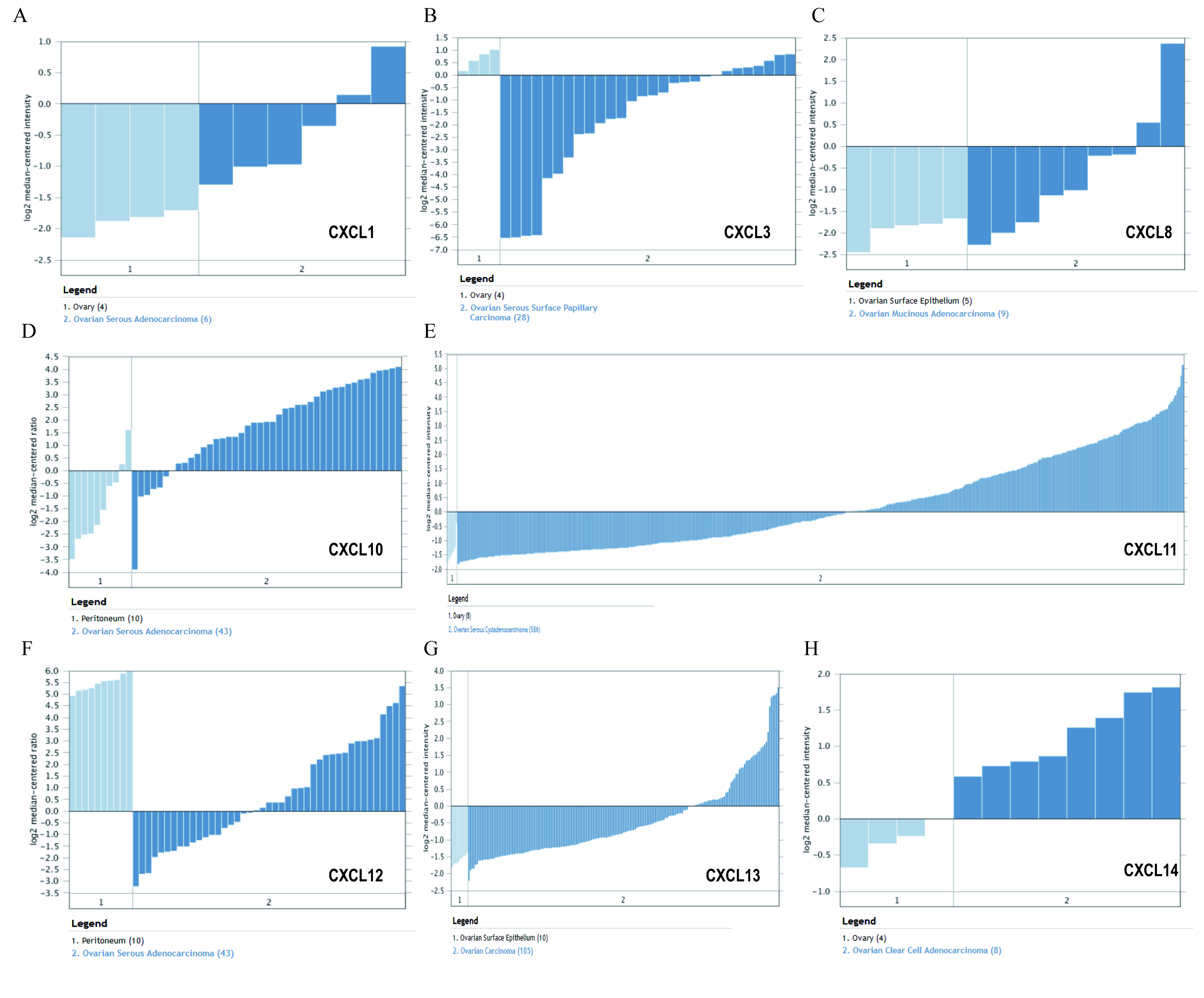
Supplementary Figure 1 Oncomine database analysis of the expression of different chemokines**

**
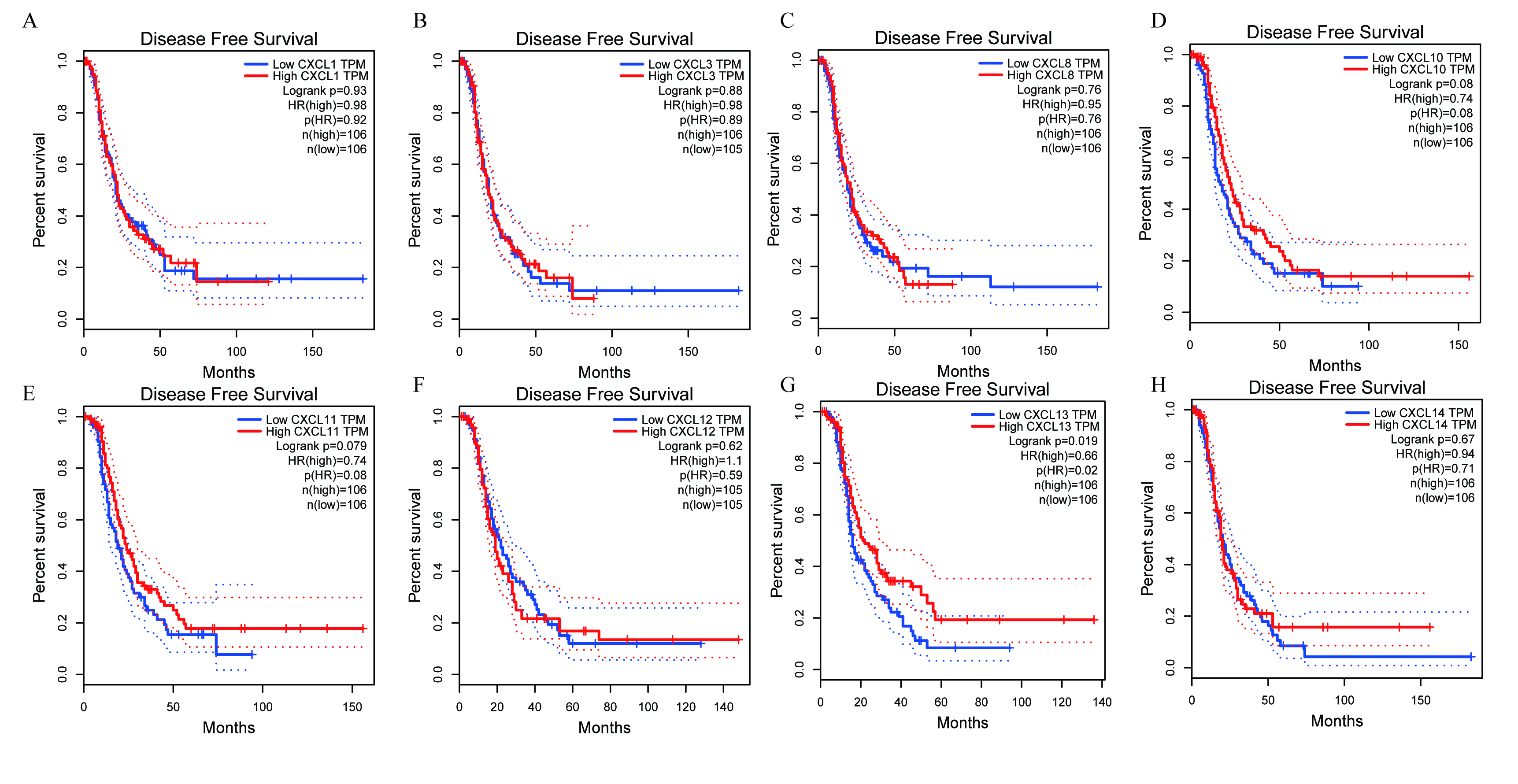
Supplementary Figure 2 The prognostic value of different expressed CXC chemokines in OC patients in the disease free survival curve**

The disease free survival curve of (A) CXCL1, (B) CXCL3, (C) CXCL8, (D) CXCL10, (E) CXCL11, (F) CXCL12, (G) CXCL13 and (H) CXCL14 in OC. All survival data are derived from TCGA data in the GEPIA database.

**
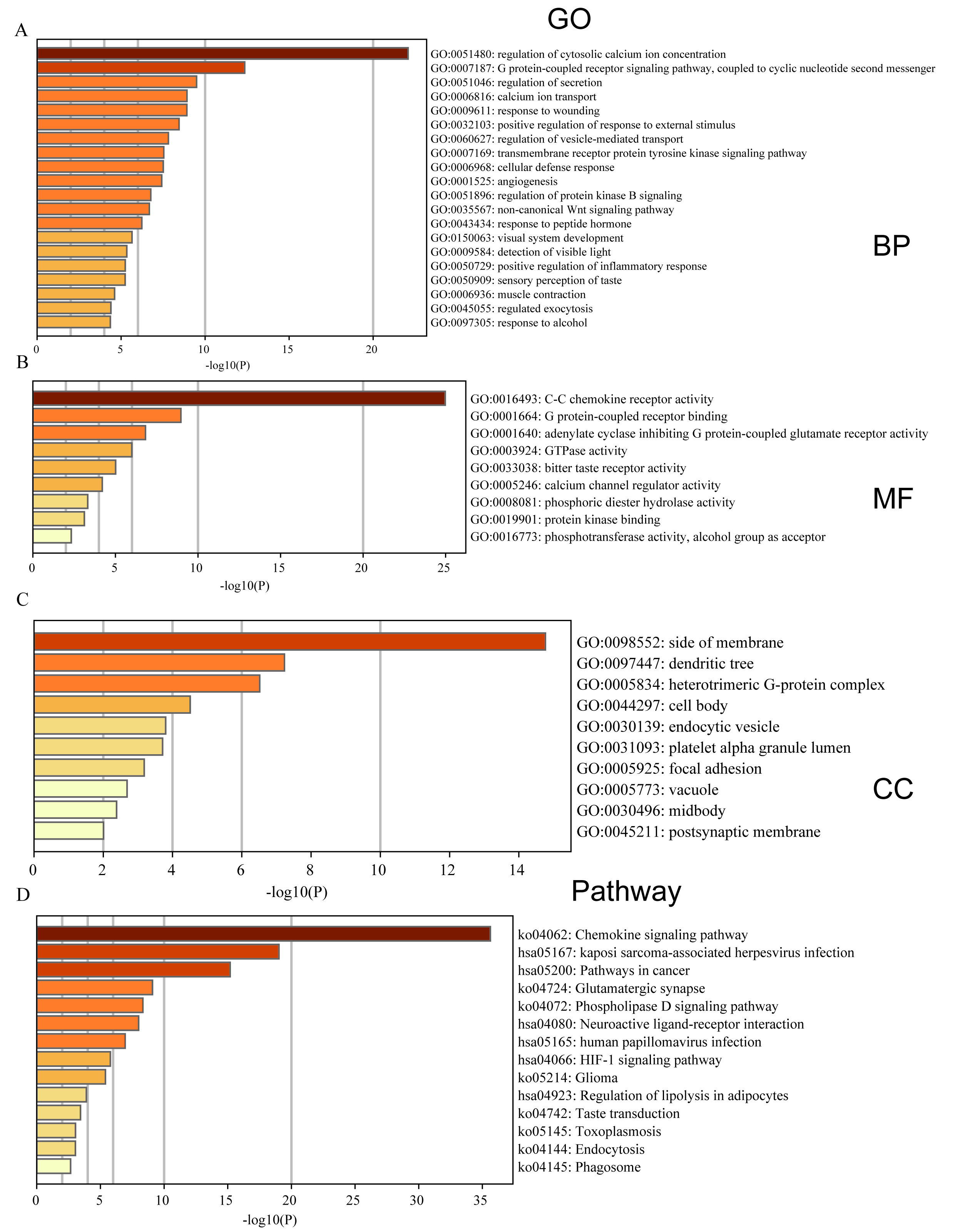
Supplementary Figure 3 GO and pathway analysis of CXC chemokines related genes using Metascape database**

In GO analysis, the enrichment results of (A) biological processes (BP), (B) molecular functions (MF), and (C) cell components (CC) were displayed, and the intensity of the enrichment results was arranged by the P value. (D). The pathway enrichment status was displayed, arranged by P value.
